# The Influence of Mirror-Visual Feedback on Training-Induced Motor Performance Gains in the Untrained Hand

**DOI:** 10.1371/journal.pone.0141828

**Published:** 2015-10-30

**Authors:** Paola Reissig, Rohan Puri, Michael I. Garry, Jeffery J. Summers, Mark R. Hinder

**Affiliations:** 1 Human Motor Control Laboratory, School of Medicine, Faculty of Health, University of Tasmania, Hobart, Australia; 2 Faculty of Health Graduate Research Program, University of Tasmania, Hobart, Australia; 3 Research Institute for Sport and Exercise Sciences, Liverpool John Moores University, Liverpool, United Kingdom; The University of Western Ontario, CANADA

## Abstract

The well-documented observation of bilateral performance gains following unilateral motor training, a phenomenon known as cross-limb transfer, has important implications for rehabilitation. It has recently been shown that provision of a mirror image of the active hand during unilateral motor training has the capacity to enhance the efficacy of this phenomenon when compared to training without augmented visual feedback (i.e., watching the *passive* hand), possibly via action observation effects [1]. The current experiment was designed to confirm whether mirror-visual feedback (MVF) during motor training can indeed elicit greater performance gains in the untrained hand compared to more standard visual feedback (i.e., watching the *active* hand). Furthermore, discussing the mechanisms underlying any such MVF-induced behavioural effects, we suggest that action observation and the cross-activation hypothesis may both play important roles in eliciting cross-limb transfer. Eighty participants practiced a fast-as-possible two-ball rotation task with their dominant hand. During training, three different groups were provided with concurrent visual feedback of the active hand, inactive hand or a mirror image of the active hand with a fourth control group receiving no training. Pre- and post-training performance was measured in both hands. MVF did not increase the extent of training-induced performance changes in the untrained hand following unilateral training above and beyond those observed for other types of feedback. The data are consistent with the notion that cross-limb transfer, when combined with MVF, is mediated by cross-activation with action observation playing a less unique role than previously suggested. Further research is needed to replicate the current and previous studies to determine the clinical relevance and potential benefits of MVF for cases that, due to the severity of impairment, rely on unilateral training programmes of the unaffected limb to drive changes in the contralateral affected limb.

## Introduction

**Fig 1:**
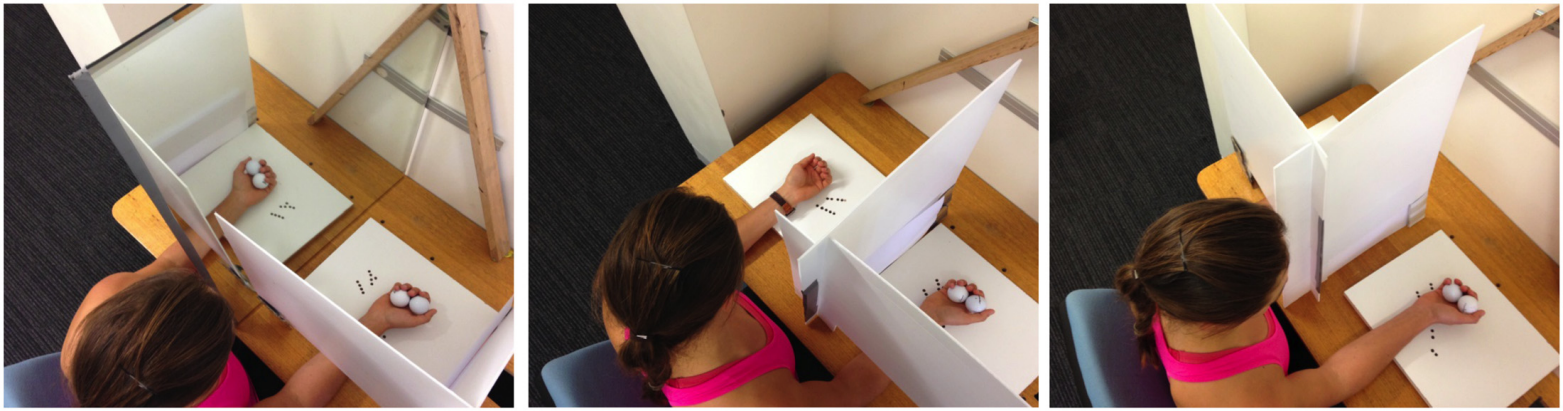
Experimental set up for the visual feedback conditions in the three training groups. Experimental set up for the visual feedback conditions in the three training groups: Mirror Vision (left), Passive Vision (middle), and Active Vision (right).

Mirror therapy is a psychophysiological technique used in the rehabilitation of individuals, who suffer from chronic regional pain syndrome or have experienced stroke or other forms of motor impairment, aiming to improve motor function or relieve pain. During mirror therapy a mirror is placed in an individual’s midsagittal plane, which participants are subsequently asked to focus on. One limb is placed in the reflective side of the mirror, and its mirror image then superimposed over the contralateral limb that is hidden behind the mirror. Once the limb in front of the mirror is moved, a visual illusion of two synchronously moving limbs is created (see Fig 1). Ramachandran and Rogers-Ramachandran [2] were the first to employ mirror-visual feedback (MVF) to alleviate phantom limb pain. Since then, mirror therapy has also been demonstrated to be beneficial in stroke rehabilitation [3, 4] and in the treatment of chronic regional pain syndrome [5]. During bilateral movement therapy within a stroke rehabilitative environment, in which a participant aims to move both arms, MVF of the unimpaired arm is superimposed over the sensed position of the impaired (paretic) arm to give the impression that the impaired limb is moving as efficiently as the unimpaired limb. Such MVF has been previously shown to elicit behavioural improvements in the impaired limb that outweigh those which occur under normal unaltered visual conditions (i.e., direct vision of the impaired limb) [3, 4, 6].

Most commonly used in conjunction with *bilateral* movement therapies within clinical settings (as described above), recent research has employed MVF in different types of *unilateral* tasks [1, 7, 8] and suggests, at least for healthy populations, beneficial behavioural effects can occur. In this context, Nojima and colleagues [1, 8] recently asked participants to practice a fast-as-possible ball rotation task with their dominant hand while providing them with different types of visual feedback. Task performance was subsequently tested in both the trained and untrained hand. The authors found significantly better test performance in the untrained hand in the group that had received MVF during training, compared to the test performance in the untrained limb for a group that had focused on the passive hand during training. Moreover, the group receiving feedback of the passive hand also exhibited significantly impaired test performance in the untrained hand compared to a group that did not actually undertake any unilateral training but instead, passively watched a third person performing the motor task with the untrained hand. This latter result was interpreted to suggest that action observation (AO) effects—consisting of either watching one’s own hand or a third person’s—may drive a substantial proportion of the performance gains exhibited in the contralateral (untrained) limb (i.e., cross-limb transfer) following unilateral training under augmented visual feedback conditions (i.e., MVF).

Cross-limb transfer (CLT) has been recognised for more than a century, and has been demonstrated for various strength and skill acquisition tasks [9–13]. Despite a number of investigations [14–16] the neural mechanisms underlying this phenomenon are not thoroughly understood. Different hypotheses have been put forward to describe the neural mechanisms of CLT and suggest that either changes in the untrained hemisphere (i.e., cross-activation hypothesis) or changes in the trained hemisphere accessible by the untrained hemisphere (i.e., bilateral access hypothesis) occur in conjunction with behavioural gains in the trained limb, underpinning successful transfer of those behavioural gains [13]. However, as described above, given that Nojima and colleagues [1] observed performance improvements in the untrained hand that were *not* contingent upon performance gains in the trained hand (nor any unilateral repeated practice at all), the idea that AO effects may be primarily responsible for CLT observed within mirror therapy was proposed. AO has been associated with the observation of *another individual* or of *oneself* performing a motor task [17, 18] and is linked with an action observation/action execution matching system in the human brain which leads to the activation of similar brain areas when observing or executing the same movement [19].

In light of our ageing society, where stroke and mobility deficits induced due to fall-related injuries is becoming increasingly common, combining unilateral motor rehabilitation programs with mirror therapy is an appealing prospect. However, in order to improve the outcome of rehabilitative programs, it is important to shed further light on the underlying mechanisms of MVF-induced behavioural gains such that these programs can be designed to facilitate those factors that critically drive the transfer process. To this end, we expanded upon Nojima and colleagues’ previous experiments [1, 8] by utilising the same motor task but including two additional visual conditions to tease apart the putative factors underlying crossed-effects in an untrained limb following motor training. Since it is most common to watch one’s own hand when undertaking fine motor tasks to ensure accurate performance, we employed a condition in which participants were provided with direct vision of the active hand during the training protocol (i.e., the most usual or ecologically valid visual feedback). In our previous studies of cross-limb transfer [11, 12] this type of feedback has been associated with cross-limb transfer of behavioural gains, and would also be expected to drive transfer in a ball rotation task if this transfer was driven by gains in the trained limb and associated cortical adaptations [13, 20]. As we do not believe hand-specific AO-effects to be the sole underlying mechanism for the current movement task, we expected visual feedback of the active hand to also elicit transfer in the untrained hand, simply as a consequence of unilateral training. However, as it is possible that AO-effects might play an additional role in modulating CLT [1, 8], we hypothesised the behavioural improvements in the untrained hand *may* be superior for the group that received MVF compared to the group that focussed on their active hand due to a combination of underlying AO and crossed-effects. Furthermore, we included a control condition, in which performance in the untrained hand was tested before and after a rest period of a commensurate amount of time to that taken for unilateral training in the other groups. We propose that such a condition would elucidate the extent to which performance improvements in the untrained limb may have occurred as a result of one-trial learning (i.e., conducting the test twice) [21] as opposed to AO or crossed-effects occurring in conjunction with gains observed in the trained hand. Indeed, test-enhanced learning has been demonstrated in a variety of cognitive and behavioural tasks and its influence on Nojima and colleagues’ paradigm cannot be assumed to be negligible.

## Methods

### Participants

Eighty members of the University of Tasmania community (mean age = 27.5 years, SD = 8.3 years, 28 men and 52 women; range 18–48 years) participated in a single session of 30 minutes duration. Six of the 80 adults (three men and three women) displayed left-hand dominance (as recorded by the Edinburgh Handedness Inventory), and all had normal or corrected-to-normal vision. The experimental procedure was approved by, and carried out in accordance with local ethical guidelines laid down by the Tasmanian Human Research Ethics Committee Network, and conformed to the Declaration of Helsinki. All participants signed an informed consent form prior to the experiment.

### Movement task

Participants were seated in a height adjustable chair with their forearms rested on a table and their palms facing upwards. Participants performed a two-ball rotation task similar to the one previously utilized by Nojima and colleagues [1, 8]. Specifically, they were asked to rotate two golf balls (43 mm diameter and 45 g) as quickly as possible in either a clockwise direction (with their right hand) or an anti-clockwise direction (with their left hand).

### Experimental design

The study investigated the effects of a motor learning task with the dominant hand on subsequent motor performance of the non-dominant hand while the nature of visual feedback provided during motor learning was manipulated. Three groups of participants practiced a fast-as-possible two-ball rotation task with their dominant hand while receiving different types of visual feedback. For a better pictorial representation of how the two balls were rotated within the palm, please refer to Fig 1A out of the Materials and Methods section of Nojima and colleagues [22]. Participants in the Active Vision (ACT: n = 20, females: 14, mean age = 28.3 years, *SD* = 8.2 years) and Passive Vision (PAS: n = 20, females: 15, mean age = 25.8 years, *SD* = 6.3 years) groups focused on the active (training) or inactive (non-training) hand, respectively, while vision of the opposite (unattended) hand was occluded with a custom built stand. For participants in the Mirror Vision group (MIR: n = 20, females: 11, mean age = 24.7 years, *SD* = 6.3 years), a mirror was placed vertically in the midsagittal plane and participants viewed a mirror reflection of their active hand. Direct vision of the inactive hand was obscured due to the positioning of the mirror; however, the mirror image of the active hand appeared superimposed on top of the obscured inactive hand. A custom-built stand, situated in the coronal plane between participants’ upper body and their active hand, also prevented a direct view of the active hand (Fig 1; the individual in this figure has given written informed consent, as outlined in PLOS ONE consent form, to publish these case details). In these three groups, participants practiced 10 blocks of 30 seconds of ball rotation. 30 seconds of rest was provided between each practice block to avoid fatigue and participants were regularly verbally encouraged to perform the task as fast as possible. Prior to, and following the training phase (total duration 10 min), participants performed the same task with their non-dominant hand for 30 seconds with similar instructions to perform the task as quickly as possible. Participants in the Control group (CON: n = 20, females: 12, mean age = 31.2 years, *SD* = 10.5 years) performed these two test blocks with their non-dominant hand, but rested between the blocks for a time period comparable to the training period in the other groups. Data of the first and last training block of the trained hand and the two test blocks of the untrained hand was collected via video recordings and stored for subsequent analysis.

### Data reduction and statistical analysis

To assess training-induced changes in performance of the dominant (trained) and non-dominant (untrained) hand, the video recordings were analysed and the number of ball rotations quantified in the pre- and post-test of the untrained hand (pre_untrained_, post_untrained_), along with the first and last block of motor learning for the trained hand (pre_trained_, post_trained_), thereafter referred to as pre- and post-performance in the trained and untrained hands. Post-performance was then normalized to pre-performance and subsequently natural log-transformed for both the trained [n_trained_ = ln(post_trained_ / pre_trained_)] and untrained [n_untrained_ = ln(post_untrained_ / pre_untrained_)] hands to avoid positive skewness that is commonly associated with normalized data.

Participants in the active training groups (ACT, PAS, MIR) who did not exhibit learning-induced performance improvements in the trained hand or did not exhibit transfer-induced performance improvements in the untrained hand were excluded from the analysis of trained and untrained hand performance. Firstly, to investigate potential differences in the trained and untrained hand at pre-test, we conducted one-way ANOVAs using pre_untrained_, pre_trained_. Subsequently, we investigated potential visual-feedback induced differences in the trained hand by conducting a one-way ANOVA on n_trained_ with groups (ACT, PAS, MIR) as a between-subject factor. Finally, to probe training-induced changes in performance of the untrained (non-dominant) hand, not only between the three training groups (i.e., ACT, PAS, MIR) but also relative to a CON group (i.e., participants that did not receive motor training with the dominant hand), we conducted a one-way ANOVA using n_untrained_.

IBM SPSS Statistics 21 (Armonk, NY, USA) was used for all analyses with the *a priori* level of two-tailed significance set at 0.05. Both normalized trained and untrained variables were tested for normality using the Kolmogorov-Smirnov test and natural log transformed (ln) in the event of a violation of the assumption of normality prior to further analysis. *Post hoc t* tests were used to examine all significant main effects and multiple comparisons corrected using the False Discovery Rate (FDR) method [23]. Partial eta-squared ($\eta_{p}^{2}$) for ANOVA’s, and Cohen’s d for student’s t tests are provided as measures of effect size and used to aid in the interpretation of inferential statistics.

## Results

All results are presented as means (*M*) ± standard deviations (*SD*), and 95% confidence intervals [CI]. There were no significant differences between the groups with regards to their age (*p* > 0.05). Table 1 shows the mean and SD for the raw number of ball rotations in each group for the trained and untrained hand at pre- and post-test.

**Table 1:** Number of ball rotations in the trained and untrained hand: Mean and SD representing the raw number of ball rotations in each group for the trained hand (N = 51) and the untrained hand (N = 59) at pre-and post-test.

	Mirror Vision		Active Vision		Passive Vision		Control Group	
	Pre	Post	Pre	Post	Pre	Post	Pre	Post
**Trained Hand**	11.9 ± 5.1	15.7 ± 4.8	12.5 ± 3.7	15.8 ± 4.5	11.6 ± 3.0	15.3 ± 2.9	n.a.	
**Untrained Hand**	14.0 ± 5.3	16.7 ± 5.6	11.5 ± 4.8	13.5 ± 4.5	11.7 ± 4.8	13.9 ± 4.8	15.2 ± 6.0	16.2 ± 6.4

### Performance of the trained hand

**Fig 2:**
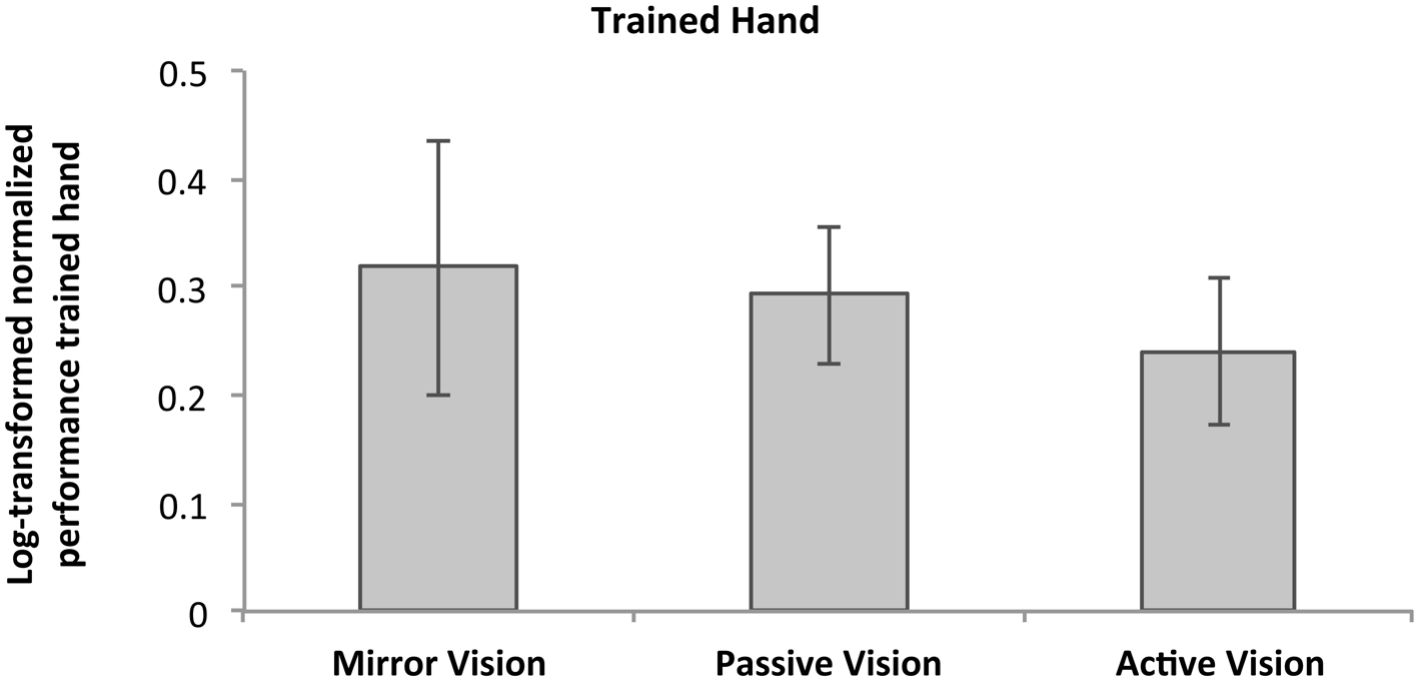
Log-transformed normalized improvement in the trained hand. Averaged normalized performance changes (log-transformed) in the trained hand for each of the three training groups (N = 51). Error bars represent 95% CI.

An initial analysis conducted on the participants who satisfied the aforementioned inclusion criteria (n MIR = 16, n PAS = 18, n ACT = 17) did not reveal a significant difference in raw performance in the trained hand at pre-test (*p* = 0.794). A subsequent one-way ANOVA also did not reveal a significant difference in motor-learning induced performance increases in the trained hand, *F*(2,51) = 0.66, *p* = 0.520, $\eta_{p}^{2}$ = 0.027, between the three active groups (MIR = 0.32 ± 0.27, [0.22, 0.42]; PAS = 0.29 ± 0.15, [0.20, 0.39]; ACT = 0.24 ± 0.16, [0.15, 0.34]). Unsurprisingly however, regardless of feedback type, participants showed substantial improvements in the trained hand (*M* = 0.28) as revealed by a significant grand mean effect, *F*(1,51) = 106.42, *p* < 0.001, $\eta_{p}^{2}$ = 0.689. Fig 2 represents natural log-transformed normalized performance gains in the trained hand for each of the training groups.

### Performance of the untrained hand

**Fig 3:**
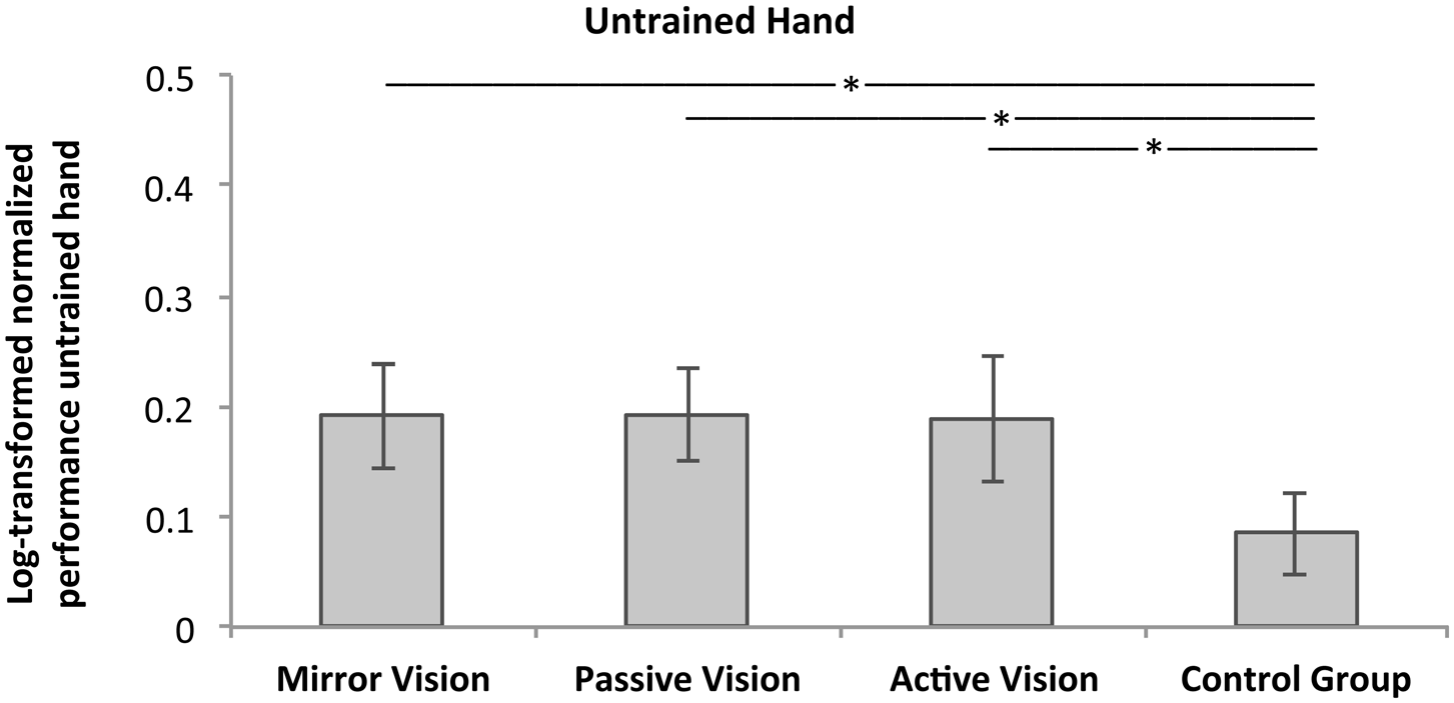
Log-transformed normalized improvement in the untrained hand. Averaged normalized performance changes (log-transformed) in the untrained hand for each of the three training groups and the control group (N = 59). Error bars represent 95% CI and * indicates FDR corrected *p <* 0.05.

An initial analysis conducted on the participants who satisfied the aforementioned inclusion criteria (n MIR = 15, n PAS = 12, n ACT = 12, n CON = 20) revealed did not reveal a significant difference in raw performance in the untrained hand at pre-test (*p* = .178). A subsequent one-way ANOVA revealed significant differences in performance gains in the untrained hand between the groups, *F*(3,59) = 6.06, *p* = 0.001, $\eta_{p}^{2}$ = 0.248. Follow up FDR- corrected t tests revealed significantly smaller performance gains for participants in the control group (CON = 0.06 ± 0.10, [0.07, 0.12]) compared to all three training groups (MIR = 0.19 ± 0.11, [0.14, 0.25]; PAS = 0.19 ± 0.10, [0.13, 0.26]; ACT = 0.19 ± 0.14, [0.13, 0.25]) (all FDR-adjusted *p’s* ≤ 0.028, all *d’s* ≥ 1.093). Additionally, none of the training groups differed between each other with respect to the extent of gains in the untrained limb, indicating that the nature of the visual feedback provided during the motor learning task did not induce a statistically significant influence on performance gains in the untrained hand (all *p’s* > 0.965). Fig 3 represents log-transformed normalized performance gains in the untrained hand for the training groups and the control group.

A similar analysis conducted on those participants who *did not* exhibit training-induced effects in the trained hand (n = 9) revealed that normalized performance in the untrained hand (log-transformed) did not differ between the training groups (0.06 ± 0.16, [-0.02, 0.14]) and the CON group (0.06 ± 0.10, [0.07, 0.12]), *F*(1,29) = 0.01, *p* = 0.940, $\eta_{p}^{2}$ < 0.001.

## Discussion

The current study aimed to determine whether MVF during unilateral motor training—in this case a fast-as-possible two-ball rotation—could result in significantly greater performance improvements in the untrained hand compared to conditions where participants focus on their active or passive hand during training, while shedding further light on the possible mechanisms mediating the transfer process. Building on the results of Nojima and colleagues [1, 8]–which appear to indicate that MVF may be beneficial in facilitating performance gains exhibited in the untrained hand—we examined the extent to which MVF facilitation can be attributed solely to action observation or whether other mechanisms such as (cortically-regulated) crossed-effects resulting from performance improvements in the trained limb may be involved once any effects of single-trial learning are taken into account. Understanding these mechanisms and the extent of their influence on transfer is essential such that mirror therapy may be utilised more widely within rehabilitative and therapeutic settings to induce the greatest possible performance gains in the untrained limb, e.g., a paretic limb following stroke.

In line with earlier research examining cross-limb transfer across different strength and skill acquisition tasks [for an overview see 9, 10, 11, 13], in the current study the degree of performance improvement after unilateral training (although seen in *both* the trained and untrained hands) was greater in the trained compared to the untrained hand. The finding that performance improvements in the trained hand did not vary between training groups indicates that any intermodal conflicts due to differences in visual feedback [24, 25] were overcome such that performance gains were elicited. Moreover, these findings are consistent with the view that performance improvements in the trained limb are only partially manifested in the untrained limb [e.g., 11–13, 20]. For those participants who exhibited performance gains in the trained hand, all feedback groups showed an increase in performance in the untrained hand (MIR: n_untrained_ = 0.19, PAS: n_untrained_ = 0.19, ACT: n_untrained_ = 0.19) that was significantly greater than the improvement observed in the control group that had not undergone training (n_untrained_ = 0.08) (*p* = 0.015). It can therefore be assumed that for the motor learning task employed in the current, as well as in previous studies [1, 8, 26, 27], the performance gains in the untrained hand were actually a consequence of prior motor training with the contralateral hand and did not simply occur as a consequence of participants performing the motor task with the untrained hand twice (i.e., pre- and post-test)–an effect previously demonstrated in a variety of cognitive and behavioural tasks [28–30].

With regards to the mechanisms mediating MVF-induced performance gains in the untrained limb, the current findings are not consistent with the idea of effector-specific AO effects as the primary underlying mechanism [1]. Rather, they are in support of the notion that training-induced improvements in the trained hand were imperative to induce performance improvements in the untrained limb. A number of lines of evidence support this view. Firstly, for all participants in *all* training groups, performance gains in the untrained hand—regardless of the nature of the visual feedback—were only apparent when training-evoked improvements in the trained limb were observed. That is, those participants who did not exhibit learning with the trained hand following the training period exhibited no gains in the untrained hand (i.e., similar improvements to the control group) (*p* = 0.940). This suggests that performance improvements in the untrained limb are contingent upon performance gains in the trained limb, a notion which is not consistent with effector-specific AO but is in accordance with different theories underlying cross-limb transfer, such as the cross-activation hypothesis [13, 20]. Secondly, if performance improvements in the untrained hand were predominantly a result of effector-specific or effector-congruent AO (i.e., facilitatory effects dependent on the congruency of the observed action), participants in our ACT group would not be expected to exhibit performance gains in the untrained hand, as observation occurred of the trained and not the untrained hand. However, they showed improvements in the untrained hand (n_untrained_ = 0.19) that did not vary significantly relative to those participants who received MVF or focussed on their passive hand during the training regime (MIR: n_untrained_ = 0.19, PAS: n_untrained_ = 0.19) (all *p’s* > 0.968) (Fig 3). According to our results, MVF-induced behavioural improvements in the untrained hand can thus not solely be attributed to effector-specific AO effects, but are also mediated, at least in part, by crossed-effects which are contingent upon training-related adaptations and performance gains in the trained limb.

We propose that MVF-induced improvements in the untrained hand are mediated, at least partially, by mechanisms similar to those underlying cross-limb transfer following unilateral training programmes in the presence of standard modes of visual feedback (i.e., watching the active hand). This proposition differs from those suggested in a number of previous studies investigating MVF-induced transfer. Based on the finding of Nojima et al. [1], two other studies [26, 27] recently argued that the neural mechanisms mediating MVF-related performance improvements in the untrained hand differed from those that mediate cross-limb transfer under more standard (veridical) visual feedback conditions. We believe that a number of behavioural and neurophysiological factors potentially contribute to the extent of cross-limb transfer exhibited after unilateral training using different visual feedback conditions. More research is thus needed to further investigate the exact contribution of those variables.

Contrary to previous reports by Nojima and colleagues [1], we did not observe statistically significant differences in the degree of performance improvement in the untrained hand depending on the type of visual feedback provided during the task [1, 8]. The current findings are, however, consistent with results from a recent study [16], in which we also proposed that a variation of visual feedback was unlikely to be the underlying driving factor behind previously reported mirror training-related behavioural improvements [see 6, 24, 25]. Despite the purported association between increases in corticospinal excitability (as a measure of plasticity changes in the motor cortex) and motor learning processes, our previous study [16] did not find increased corticospinal excitability facilitation in the ipsilateral motor cortex in the MVF condition when compared to more standard visual feedback conditions (i.e., watching the active or the passive hand). It was thus concluded that the unilateral execution of the movement itself represented the more important mechanism underpinning MVF-induced gains in the untrained hand, with AO-effects potentially being manifested concurrently to a lesser degree.

Alternatively, it is conceivable that the inconsistency in findings between the current study and Nojima and colleagues [1] may reflect subtle differences in the experimental setup. Specifically, participants in the mirror group in the current study were *only* able to see the mirror image of the active hand (superimposed over the inactive hand), whereas in Nojima and colleagues’ experiments [1, 8] participants were permitted peripheral vision of the active hand as well as its mirror image. The cross-activation hypothesis of cross-limb transfer [13] is predicated upon the fact that unilateral tasks are associated with bilateral cortical activation, e.g., an increase in unilateral force leads to an excitability increase in the projections to the opposite limb [31]. Accordingly, allowing people to view both ‘hands’ (i.e., the active hand and the mirror image—as was the case in Nojima et al’s studies) may have led to more pronounced changes in the M1 ipsilateral to the active hand and may subsequently have led to greater performance increases when compared to the MVF condition in the current study, where participants only saw a single limb. This view is also supported by previous research investigating the underlying neural mechanisms of MVF [32–34]. Specifically, Fritzsch and colleagues [34] argued that the production of additional ipsilateral activation in M1 from MVF might have been due to the availability of vision of the mirror *and* the active hand during task execution. However, as we did not attain any neurophysiological measures, nor test conditions in which one or both hands were visible, we are unable to determine whether this proposition holds true in the current experiment.

In considering a previous study by Nojima and colleagues [22], which found behavioural improvements after AO to be dependent on and positively correlated to the degree of kinaesthetic illusion elicited by the AO, it is conceivable that our MIR condition failed to induce a significant enough kinaesthetic illusion in the untrained hand such that this condition failed to elicit performance gains that were superior to those observed in the other feedback groups. One of the potential limitations to the conclusions drawn from the current study is the substantial inter-participant variability observed with respect to performance, both prior to, and following motor training, possibly suggesting a high degree of task complexity. Moreover, the degree of learning observed over the course of the training was very low (i.e., an increase of only 2–3 ball rotations within the 30 s period), which is likely to have resulted in any subtle differences in the extent of learning (in either the trained or transfer hand), eliciting due to changes in feedback, remaining undetected. An associated consideration is that, consistent with Nojima and colleagues [1], we used the same sized balls for all participants. This may have made the task easier for some individuals, and harder for other, depending on whether the ball diameter was appropriate for their palm size. In addition, we used a set of balls that differed in terms of their size and weight (43 mm diameter and 45 g) compared to those used in previous studies (30 mm diameter and 10g weight, see 1, 8, 26, 27). Such a difference may have accounted for the lower baseline performance in both the trained (mean = 15 rotations) and untrained (mean = 14 rotations) hand observed in the current study compared to the previously mentioned studies (Nojima [1]: approx. 21 rotations over a 30 second period; von Rein [26]: 43 rotations over a 1 minute period), further hindering sufficient performance gains and possible transfer. Finally, errors in coordination (i.e., ‘slips’ or ball drops), despite being corrected for quickly, could substantially affect the number of ball rotations achieved in the short 30 second test period resulting in large variability. We propose that future studies employ an array of tasks of varying complexity (e.g., equipment size adjusted to hand size) that are sensitive enough to evaluate whether MVF is more effective for certain types of tasks. Moreover, we recommend evaluating motor performance throughout the entire training period [26, 27] as opposed to only assessing pre- and post-training measures, thus enabling more accurate conclusions to be drawn about participants’ change in performance over time. Finally, in light of previous findings [22], we suggest assessment of the degree of kinaesthetic illusion elicited across the different feedback groups, as such illusory effects might be an important factor explaining and determining the success of MVF-based interventions.

In conclusion, the present study does not support previous suggestions that MVF has the potential to increase the extent of training-induced performance changes in the untrained hand following unilateral training above and beyond those observed for other types of feedback. Furthermore, the data are consistent with the notion that CLT effects are mediated, at least in part, by neural adaptations [20] that occur in conjunction with behavioural gains in the trained limb, and AO, in contrast, appears to play not as significant a role as suggested by recent reports [1]. Further research is needed to replicate and expand upon the current and previous studies to determine clinical relevance, especially for cases in which rehabilitation using bilateral movement therapies is not possible due to the severity of the impairment, and thus increasing the importance of unilateral training programs.

## Supporting Information

S1 File Raw data for all 80 participants: Raw pre- and post-performance in the trained and untrained hands for the entire sample size. (XLSX)
